# The effect of a ketone monoester drink on blood glucose and substrate oxidation in adults with metabolic syndrome and matched controls

**DOI:** 10.14814/phy2.70676

**Published:** 2025-11-28

**Authors:** Austin J. Graybeal, Ryan S. Aultman, Caleb F. Brandner, Anabelle Vallecillo‐Bustos, Abby T. Compton, Sydney H. Swafford, Ta’ Quoris A. Newsome, Ryan R. Porter, Jon Stavres

**Affiliations:** ^1^ Department of Kinesiology, Harris College of Nursing and Health Sciences Texas Christian University Fort Worth Texas USA; ^2^ School of Health Sciences, College of Education, Health, and Human Services Kent State University Kent Ohio USA; ^3^ Department of Health and Human Physiology, College of Liberal Arts and Sciences University of Iowa Iowa City Iowa USA; ^4^ School of Kinesiology & Nutrition, College of Education and Human Sciences University of Southern Mississippi Hattiesburg Mississippi USA; ^5^ School of Medicine, University of Mississippi Medical Center University of Mississippi Jackson Mississippi USA; ^6^ Department of Kinesiology, Nutrition, and Health, College of Education, Health, and Society Miami University Oxford Ohio USA

**Keywords:** energy expenditure, ketone ester, ketosis, metabolic syndrome, metabolism, substrate utilization

## Abstract

This study aimed to evaluate the acute metabolic effects of oral ketone esters (KE) in individuals with and without metabolic syndrome (MetS) on resting energy expenditure (REE), respiratory exchange ratio (RER), and substrate utilization using respiratory gas exchange. Eight participants with MetS and 8 without MetS matched for age, race, and ethnicity completed a randomized, single‐blind, placebo‐controlled crossover study. Participants underwent a cardiometabolic screening to confirm MetS, followed by two experimental trials. Respiratory gases were measured for 15 min at baseline and 45 and 105 min after a randomly assigned KE or placebo beverage. Following KE ingestion, RER, carbohydrate oxidation (CarbOx), and blood ketones increased significantly, while blood glucose and fat oxidation (FatOx) decreased significantly, irrespective of group. The MetS group showed higher βHB responses to the KE condition than their matched controls, but blood glucose reductions were comparable between groups. Substrate oxidation was similar between the MetS and matched control groups across conditions. Irrespective of MetS status, oral KE acutely increase CarbOx and RER, while concurrently reducing FatOx, without altering REE, suggesting that substrate utilization shifts toward greater CarbOx following KE. Exogenous ketosis may be a promising nonpharmacological strategy to improve metabolic function in individuals with or at risk for MetS.

## 1 INTRODUCTION

Metabolic syndrome (MetS) is a precursory chronic health condition characterized by an assemblage of risk factors including central obesity, dyslipidemia, hypertension, and impaired glucose regulation (Grundy et al., 2005). Collectively, these risk factors increase the likelihood of developing type 2 diabetes (T2D) and cardiovascular disease (CVD), reinforcing MetS as a prevalent and rising public health concern (Han & Lean, 2016; Liang et al., 2023). While insulin resistance and mitochondrial dysfunction represent well‐established characteristics of MetS (Prasun, 2020; Ruderman et al., 2013), these elements are thought to underpin other distinctive features of MetS such as impaired regulation of substrate utilization pathways, impacting the ability to switch between available or preferred substrates (i.e., carbohydrates and fats) in response to specific physiological demands (Goodpaster & Sparks, 2017). This dysregulation, which is often accompanied by reduced fat oxidation (FatOx) at rest and elevated fasting glucose levels, appears to contribute to the progression of MetS by advancing the cardiometabolic abnormalities that facilitate MetS development (Smith et al., 2018). Given the limited adherence rates for most lifestyle interventions in preventing chronic disease (De Bacquer et al., 2022), and the limited or inaccessible treatment options for MetS, non‐pharmacological solutions for MetS beyond traditional diet and exercise interventions are critical in slowing the increasing prevalence of MetS.

Ketosis, a metabolic state in which ketone bodies become a primary energy substrate, has been shown to improve glycemic control and alter substrate utilization (Stubbs et al., 2017). Traditionally, ketosis is achieved through prolonged fasting or adherence to a ketogenic diet, both of which are limited by low adherence and sustainability. Alternatively, oral ingestion of exogenous ketone esters (KE) offers a practical means of inducing acute hyperketonemia without dietary restriction (Graybeal et al., 2025). KE supplementation rapidly elevates circulating β‐hydroxybutyrate (βHB), which can be oxidized by metabolically active tissues and may serve as an efficient alternative to fatty acids for energy production (Veech, 2014), especially for those with the metabolic dysfunctions that accompany MetS.

Current theories propose that KE ingestion can acutely lower blood glucose and shift substrate utilization toward greater carbohydrate oxidation (CarbOx) and reduced FatOx (Fernández‐Verdejo et al., 2023), which may be particularly beneficial for individuals with MetS who often exhibit impaired insulin sensitivity and reduced mitochondrial efficiency (Prasun, 2020; Ruderman et al., 2013). Yet, the acute metabolic effects of KE ingestion in individuals with MetS remain poorly understood, particularly in the context of respiratory gas exchange measurements. Therefore, the purpose of this study was to evaluate the acute effects of oral KE ingestion on metabolic responses, specifically resting energy expenditure (REE), respiratory exchange ratio (RER), and substrate oxidation, in individuals with and without MetS. We hypothesized that KE ingestion would increase REE, RER, and CarbOx while reducing FatOx, with more pronounced effects in individuals with MetS due to their underlying metabolic impairments.

## 2 MATERIALS AND METHODS

### 2.1 Experimental design and outcome variables

**FIGURE 1:**
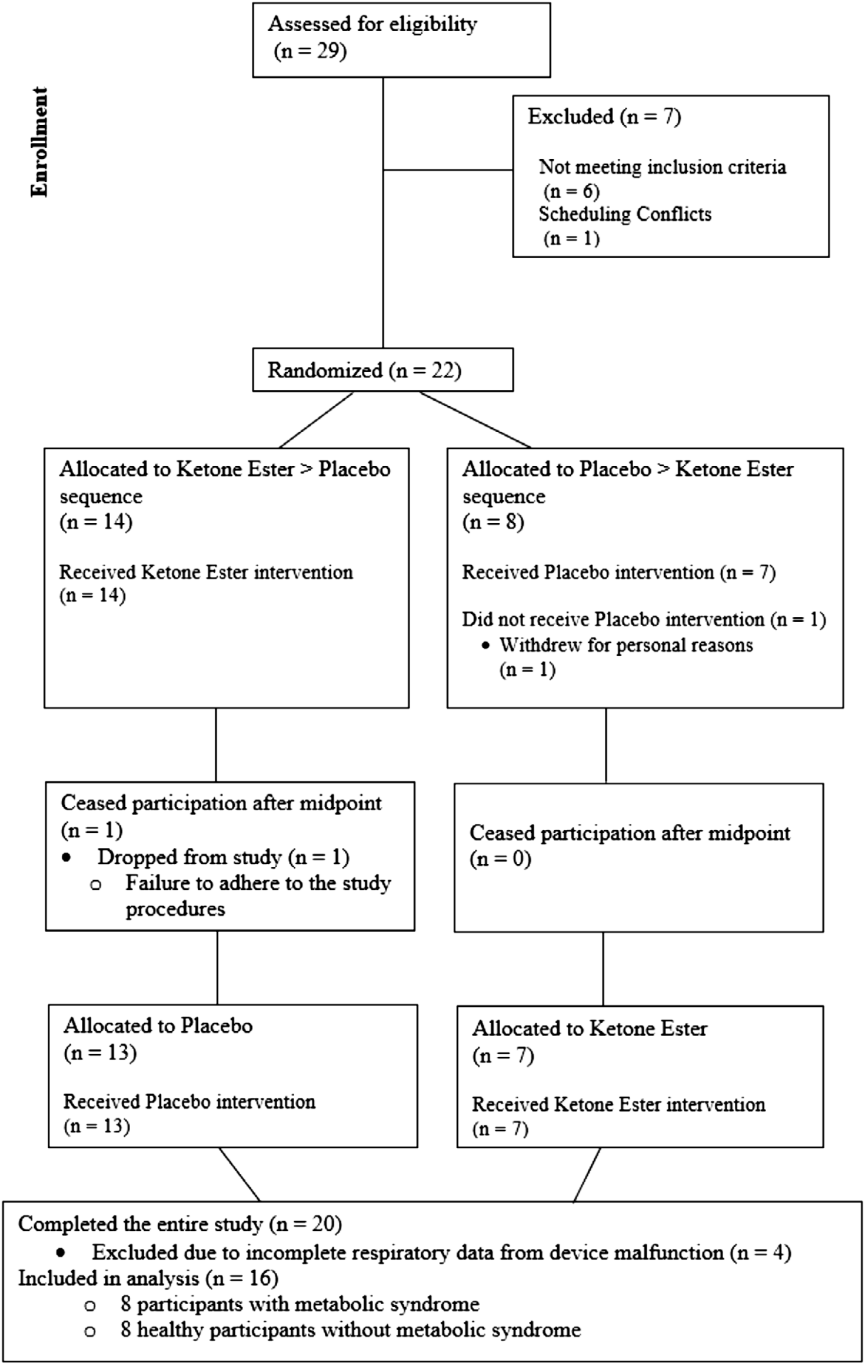
Study CONSORT diagram.

This randomized, single‐blind, placebo‐controlled, matched‐pairs crossover study represents a secondary analysis conducted in parallel with a broader line of investigation (Graybeal et al., 2025; Stavres et al., 2025). The primary outcomes of the present analysis—REE and substrate utilization assessed via respiratory gas exchange—are unique to this study and do not overlap with the primary endpoints of the parent project. Only a limited set of secondary variables, including descriptive characteristics and capillary concentrations of beta‐hydroxybutyrate (βHB) and blood glucose, are shared across related investigations (Graybeal et al., 2025; Stavres et al., 2025). This trial was prospectively registered at ClinicalTrials.gov (NCT05651243), and the full CONSORT diagram is provided in Figure 1. This study was conducted according to the guidelines laid down in the Declaration of Helsinki and all procedures involving human subjects/patients were approved by the University of Southern Mississippi Institutional Review Board (IRB# 22‐877). Written informed consent was obtained from all participants prior to participation.

### 2.2 Participants

Participant recruitment, matching procedures, and a comprehensive list of exclusion criteria are detailed elsewhere (Graybeal et al., 2025; Stavres et al., 2025). Briefly, individuals aged 18–55 years were prospectively recruited for the intervention group and were eligible if they met the Adult Treatment Panel III (ATP‐III) criteria for MetS (Grundy et al., 2005), defined by the presence of at least three of the following five risk factors: (i) waist circumference (WC) ≥88 cm for females or ≥102 cm for males; (ii) high‐density lipoprotein cholesterol (HDL‐C) <50 mg/dL for females or <40 mg/dL for males; (iii) triglycerides (TRG) ≥150 mg/dL; (iv) systolic blood pressure (SBP) ≥130 mmHg or diastolic blood pressure (DBP) ≥85 mmHg; and (v) fasting blood glucose (FBG) ≥100 mg/dL.

The goal of the project was to verify eligibility for the MetS group and subsequently recruit participants for the non‐MetS group (<3 NCEP ATP III risk factors and without abdominal obesity), who were matched to a participant with MetS based on sex, age, race, and ethnicity. We ultimately achieved this goal by recruiting 10 participants per group, who were matched by sex (6 females, 4 males per group), age, race, and ethnicity. However, two participants from each group (2 female participants with MetS; 1 female and 1 male participant without MetS) had incomplete respiratory gas data across multiple timepoints due to device malfunction. Thus, these participants were excluded from this evaluation, resulting in one less male and one additional female in the non‐MetS group. However, each group remained evenly matched for race (4 White, 4 Black participants per group), ethnicity (1 White Hispanic participant per group), and age (MetS: 31 ± 13 years; non‐MetS: 29 ± 10 years; *p* = 0.560) in accordance with the guidelines of the American Aging Association (Geifman et al., 2013). Moreover, using an expected small‐to‐moderate effect size and correlation (*f* = 0.35; *r* = 0.50), it was determined that a total of 16 participants would yield 83.7% power for within‐between interactions using mixed‐models (2 groups; 3 repeated measurements).

Thus, the final sample consisted of 16 participants: 8 in the MetS group (5 M, 3 F) and 8 in the non‐MetS group (4 M, 4 F), matched by age, race, and ethnicity. Group‐specific characteristics are presented in Table 1. After inclusion, eligible participants were randomly assigned to begin with either the KE or placebo condition (KE → Placebo or Placebo → KE). After completing the first trial, participants underwent a washout period of at least 4 days before completing the alternate condition. An online randomization tool (http://www.random.org/) was used to generate the randomization sequence and participants were allocated to the next available sequence upon confirmation of participation (Graybeal et al., 2025; Stavres et al., 2025).

**TABLE 1:** Participant demographics.

	MetS	Non‐MetS
Sex (M/F)	5/3	4/4
Race (W/AA)	4/4	4/4
Ethnicity (HW/NHW)	1/7	1/7
Medication use^a^	3	0
Age (yrs)	31 ± 13	29 ± 10
Height (cm)	171.1 ± 11.5	168 ± 12.4
Weight (kg)	88.7 ± 25.1	64.4 ± 14.0*
BMI (kg/m^2^)	29.9 ± 6.3	22.5 ± 2.2*
Waist circumference (cm)	101.2 ± 13.9	79.1 ± 9.9*
BF (%)	33.8 ± 7.3	23.8 ± 5.0*
Fat mass (kg)	32.1 ± 12.7	15.2 ± 3.6*
Fat‐free mass (kg)	63.7 ± 18.5	49.3 ± 11.9
SBP (mmHg)	126 ± 9	114 ± 14
DBP (mmHg)	94 ± 13	77 ± 11*
HDL‐C (mg/dL)	37 ± 13	49 ± 12
TRG (mg/dL)	212 ± 196	76 ± 32*
FBG (mg/dL)	101 ± 13	87 ± 5*

*Note*: Data presented as mean ± standard deviation or as the sample size (n/n).

Abbreviations: B, Black; BF, body fat percentage; DBP, diastolic blood pressure; F, female; FBG, fasting blood glucose; HDL, high‐density lipoprotein cholesterol; HW, Hispanic White; M, male; MetS, metabolic syndrome group; NHW, Non‐Hispanic White; Non‐MetS, no metabolic syndrome group; SBP, systolic blood pressure; TRG, triglycerides; W, White.

^a^ Using medications to treat or control hypertension, hyperglycemia, and/or dyslipidemia.

* Significantly different between groups at *p* ≤ 0.050.

### 2.3 Procedures

The study procedures are presented elsewhere (Graybeal et al., 2025; Stavres et al., 2025) but are summarized hereafter with additional information specific to this secondary analysis. Testing was conducted on three separate occasions: the initial visit, which included a cardiometabolic screening to confirm eligibility, followed by two experimental visits, each separated by a minimum 4‐day washout period. For the cardiometabolic screening, participants reported to the laboratory following at least 8 h of abstention from food, beverages, supplements, and medications (i.e., overnight fast), and at least 24 h of exercise abstention, both of which were confirmed by an investigator. After reporting to the facility, participants had their height measured using a stadiometer, weight measured by a calibrated digital scale, WC using a flexible aluminum tape measure following established procedures (Graybeal, Brandner, Compton, et al., 2024; Graybeal, Brandner, & Tinsley, 2023), and body composition analysis via bioelectrical impedance spectroscopy (Graybeal, Brandner, & Stavres, 2024). For body composition assessments, participants were instructed to stay adequately hydrated, but not overhydrated, the evening prior to their visit. Participants were then seated in an upright position for a minimum of 5 min prior to the assessment of SBP and DBP using an automated blood pressure monitor. Finally, 40 μL of capillary blood was collected via fingerstick using a heparin‐lined capillary pipette to assess fasting blood lipids and glucose with a validated capillary blood analyzer (Cholestech LDX, Abott, Abbott Park, IL) (Graybeal, Brandner, Compton, et al., 2024; Stavres et al., 2023).

For the second and third visits, participants completed either the KE or placebo trial in accordance with their randomly assigned sequence. For both experimental trials, participants arrived at the laboratory following an ≥8 h overnight fast, ≥24 h abstention from exercise, and adherence to their habitual dietary intake, which was confirmed between trials by an investigator through the assessment of a 24‐h dietary record on the day prior to each experimental trial. Following their arrival, participants were instructed to void their bladder to minimize movement and avoid equipment removal during the trial. Weight was then measured to confirm the absence of weight fluctuation between trials. Afterwards, participants assumed a semi‐recumbent position (legs parallel to the ground and the torso elevated to approximately 40°), during which baseline respiratory gases, blood ketones (i.e., βHB), and blood glucose were collected. βHB and blood glucose were assessed at each timepoint, in duplicate, using a validated point‐of‐care βHB and blood glucose monitor (GK+, Keto‐Mojo, Napa, CA, USA) (Moore et al., 2021). Once all baseline assessments were completed, participants ingested their randomly assigned treatment within 2 min, and respiratory gases, βHB, and blood glucose were re‐evaluated at 45 and 105 min post‐ingestion. Duplicate measurements of βHB and blood glucose were averaged to produce the final estimate used for analysis. The same procedures were used for each visit, with the only difference between the experimental trials being the ingestion of either the KE or placebo.

### 2.4 Study treatment preparation and dosing protocol

The complete preparation and dosing protocol has been described in prior investigations (Graybeal et al., 2025; Stavres et al., 2025). For the KE treatment, participants ingested 282 mg/kg body mass of a commercially available ketone monoester (∆G Tactical, T∆S Global Inc., Orlando, FL; PubChem CID: 70686887) (Stubbs et al., 2017) diluted in filtered water to create a 250 mL solution. To improve palatability and flavor masking, 2 tablespoons of stevia leaf extract were added to the solution. In the placebo condition, participants ingested a volume‐ and taste‐matched solution containing filtered water, a bittering agent (Bitrex, Veranova L.P., Edinburgh, Scotland; PubChem CID: 23673652), 2 tablespoons of stevia leaf extract (PubChem CID: 6918391), and 2 g of cellulose powder (PubChem CID: 16211025). After fully consuming each treatment within 2 min, participants drank an additional 100 mL of filtered water—poured into the original treatment container—to ensure complete ingestion and to help eliminate any residual taste.

### 2.5 Resting energy expenditure and substrate oxidation

REE and substrate utilization were measured from respiratory gases via indirect calorimetry (True One 2400, Parvo Medics, Salt Lake City, UT) with a Hans‐Rudolph silicone face mask (COSMED USA Inc., Chicago, IL) using well‐established measurement guidelines (Cooper et al., 2009). Due to prior assessments conducted during the visit, participants remained motionless in a semi‐recumbent position for ≥20 min prior to the first respiratory gas measurement, which occurred in a climate‐controlled room (Compher et al., 2006; Graybeal, Kreutzer, et al., 2023; Tinsley et al., 2018). After the ≥20 min resting period, respiratory gases were measured for 15 min (baseline). After baseline respiratory gas measurements were completed, participants consumed their randomly assigned KE or placebo beverage, and respiratory gases were measured again at 45‐ and 105‐ min post‐ingestion for a total of 125 min of monitoring in a semi‐recumbent position, which includes the time between baseline measurements and the end of the final testing period. Notably, these measurement timepoints (baseline, 45 min, 105 min) were selected to represent the most consistent, uninterrupted, and motionless periods of the parent study's design (Graybeal et al., 2025; Stavres et al., 2025), with the first 5 min of the 15 min respiratory gas collection omitted to ensure the most stable resting measurements (Tinsley et al., 2018). Gas and flow calibrations were conducted daily prior to all assessments (Compher et al., 2006; Tinsley et al., 2018). RER was calculated as the rate of carbon dioxide production (V̇CO2) divided by the rate of oxygen consumption (V̇O2). Ventilation rate (VE) was measured as the product of the tidal volume and respiratory rate. REE was calculated using the equations from Mehta et al. (2015) converted to kcals/min, and FatOx and CarbOx were calculated using the equations from Frayn (1983)—each of which assumes no protein oxidation.

$$\mathrm{REE}\;\left(\text{kcal}/d\right):\left[3.941\left(\dot{V}\mathrm{CO}2/\mathrm{RER}\right)+1.106\left(\dot{V}\text{CO2}\right)\right]\times 1440$$

$$\text{FatOx}\;\left(g/\min\right)=\left(1.67\times \dot{V}\text{O2}\right)-\left(1.70\times \dot{V}\text{CO2}\right)$$

$$\text{CarbOx}\;\left(g/\min\right)=\left(4.56\times \dot{V}\text{CO2}\right)-\left(3.21\times \dot{V}\text{O2}\right)$$

### 2.6 Statistical analyses

Descriptive characteristics between groups were compared using Mann–Whitney *U* tests, and differences in baseline blood ketones, blood glucose, and body mass between treatments were assessed using paired *t*‐tests. Within, between, and interaction effects for respiratory gas and capillary blood responses were analyzed using mixed models with simple effects post hoc tests. Effect sizes are presented as *η* ^2^_p_ for omnibus models and as Cohen's d (with 95% confidence intervals [95%CI]) for simple effects tests. Group mean imputation (based on MetS or non‐MetS classification) was applied only for a single participant to replace a missing baseline respiratory gas value in the placebo condition due to a power outage and was solely used for descriptive baseline comparisons. Missing data was also handled by using one value for blood ketone and glucose measurements when averaging the duplicate values was not possible. Normality was confirmed via visual inspection of Q‐Q plots, histograms, and box plots. Significance was accepted at *p* ≤ 0.050. Similar to what has been reported in prior analyses stemming from the larger project (Graybeal et al., 2025), there were no significant effects of order on condition, group, or interaction effects for any variable (all *p* ≥ 0.075).

## 3 RESULTS

### 3.1 Participant characteristics and blood ketone and glucose responses

Individuals in the MetS group had significantly higher weight (*p* = 0.040), BMI (*p* = 0.002), WC (*p* = 0.004), body fat percentage (*p* = 0.007), fat mass (*p* < 0.001), DBP (*p* = 0.021), TRG (*p* = 0.010), and FBG (*p* = 0.027) compared to the non‐MetS participants (Table 1). No other descriptive characteristics were significantly different between groups (all *p* ≥ 0.072). Importantly, participants' baseline βHB (all *p* ≥ 0.196), blood glucose (all *p* ≥ 0.075), and body mass (all *p* ≥ 0.248) were not significantly different between trials (KE vs. placebo).

**FIGURE 2:**
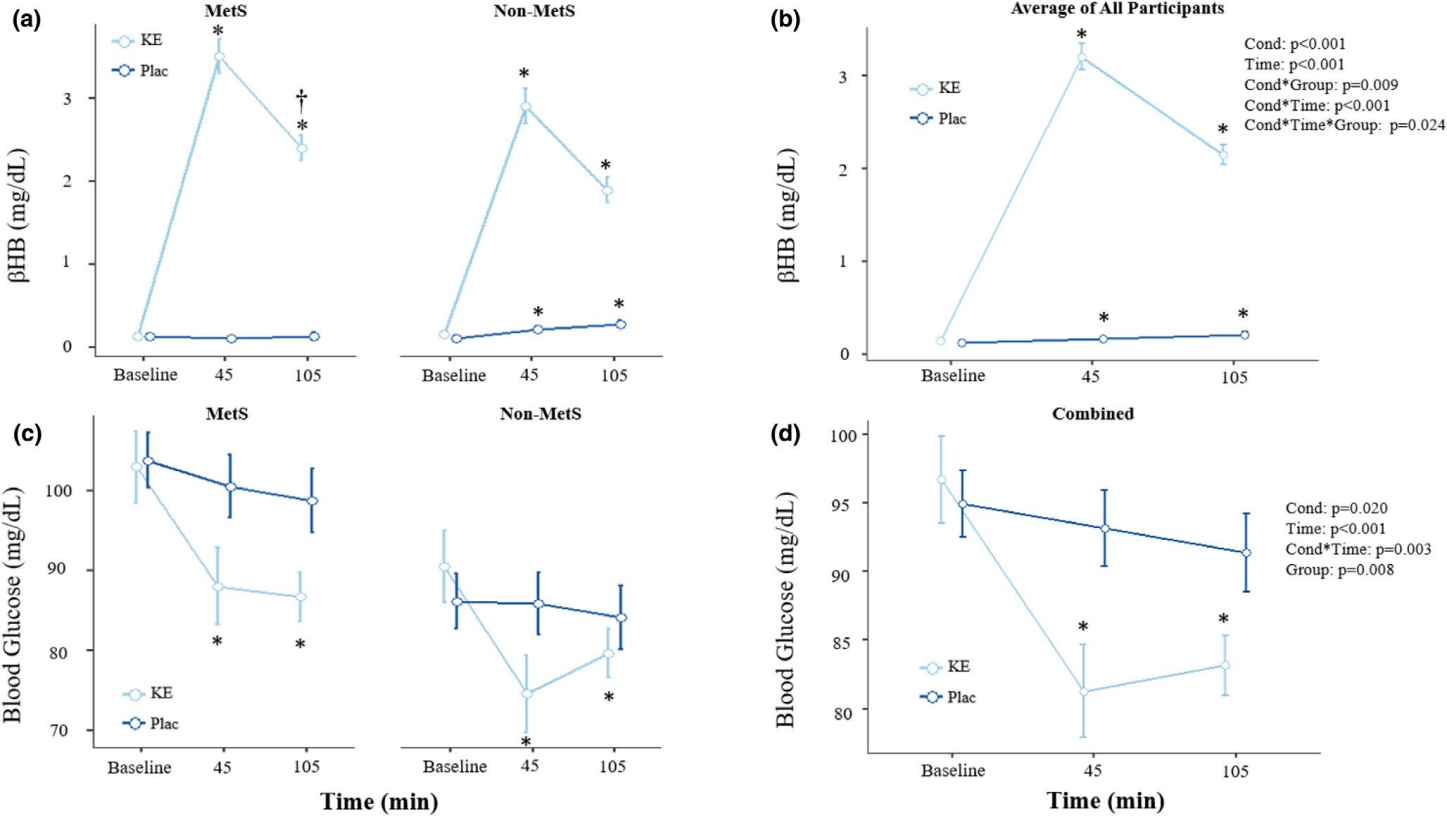
Blood ketone (βHB) (a, b) and blood glucose (c, d) concentrations compared across time and between the ketone (KE) and placebo (Plac) conditions for the independent metabolic syndrome (MetS) and non‐MetS groups (left column), as well as the average response across all participants (right column). Each point and corresponding error bar represent the mean ± standard error for each timepoint. *, significant difference from baseline within condition; † significant difference between MetS and non‐MetS groups; (*p* ≤ 0.050).

Results comparing βHB and blood glucose responses between conditions by group are illustrated in Figure 2a–d. Significant condition by time (*p* < 0.001; *n* ^2^_p_ = 0.94), condition by group (*p* = 0.009; *n* ^2^_p_ = 0.40), and condition by group by time (*p* = 0.024; *n* ^2^_p_ = 0.23) interactions were observed for βHB. During the KE trial, βHB increased across all time points in the combined, MetS, and non‐MetS groups (Figure 2a,b; all *p* < 0.001). Moreover, βHB was significantly higher for the MetS than the non‐MetS group at 105 min post ingestion (*p* = 0.030; d: 1.21; d^95%CI^: 0.12, 2.27). For the combined and non‐MetS groups, βHB significantly increased during the placebo condition beginning at 45 min post‐ingestion (combined: *p* = 0.021; non‐MetS: *p* < 0.001), but only one participant reached a βHB concentration ≥0.50 mmol/L (105 min post‐ingestion) indicating the absence of ketosis at the group level. There were no significant changes in βHB for the MetS group during the placebo condition (all *p* ≥ 0.410), and no MetS participant obtained a βHB concentration ≥0.50 mmol/L. Significant condition by time interactions were observed for blood glucose (*p* = 0.003; *n* ^2^_p_ = 0.34), where blood glucose decreased from 45 to 105 min post KE in the combined (Figure 2d; all *p* < 0.001), MetS (Figure 2c; all p < 0.001) and non‐MetS groups (Figure 2c; all *p* < 0.007). No differences from baseline blood glucose were observed during the placebo trial for any group (all *p* ≥ 0.292).

### 3.2 Resting energy expenditure and substrate oxidation

**FIGURE 3:**
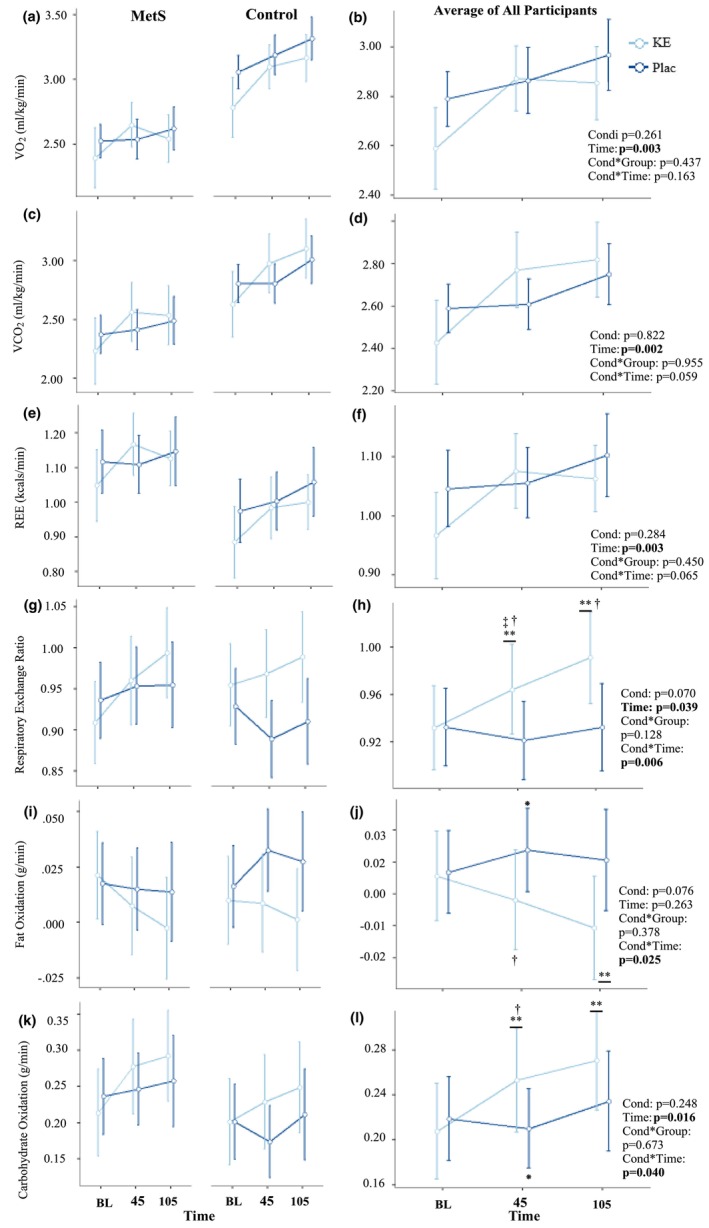
Changes in relative oxygen (V̇O2) consumption (a, b), relative carbon dioxide (V̇CO2) production (c, d), REE (e, f), respiratory exchange ratio (RER) (g, h), and fat (i, j) and carbohydrate oxidation (k, l) compared across time and between the ketone ester (KE) and placebo (Plac) conditions for the independent metabolic syndrome (MetS) and non‐MetS groups (left column), as well as the average response across all participants (right column). Each point and corresponding error bar represent the mean ± standard error for each timepoint. ** significant difference from baseline within condition; † significant difference between conditions; ‡ significant difference from 105 min within condition; * significant difference from 105 min between conditions; (*p* < 0.050).

Metabolic responses for each group following the ingestion of both conditions are presented in Figure 3a–l. No main nor condition by time interactions were observed for relative V̇O_2_, V̇CO_2_, VE, or REE (Figure 3a–f) throughout the trial (all *p* ≥ 0.059), and no between‐group differences or interactions were observed (all *p* ≥ 0.137).

Significant condition by time interactions (Figure 3h; *p* = 0.039; *n* ^2^_p_ = 0.21) were observed for RER. Following the KE, RER was higher at 45 (*p* = 0.034; d_z_: 0.57; d^95%CI^: 0.03, 1.09) and 105 min (*p* = 0.005; d_z_: 0.81; d^95%CI^: 0.23, 1.37) compared to baseline, and higher at 105 compared to 45 min (*p* = 0.047; d_z_: 0.56; d^95%CI^: 0.02, 1.08). RER was also higher at 45 (*p* = 0.020; d_z_: 0.58; d^95%CI^: 0.04, 1.11) and 105 min (*p* = 0.036; d_z_: 0.59; d^95%CI^: 0.05, 1.11) after ingestion of the KE compared to 45 and 105 min after ingestion of the placebo, respectively.

Significant condition by time interactions were observed for FatOx (Figure 3j; *p* = 0.025; *n* ^2^_p_ = 0.23), where FatOx was lower than baseline 105 min after ingestion of the KE (*p* = 0.016; d_z_: −0.67; d^95%CI^: −1.21, −0.12). Moreover, FatOx was lower at 45 min after ingestion of the KE compared to 45 min after the placebo (*p* = 0.044; d_z_: −0.55; d^95%CI^: −1.07, −0.01), and 105 min after ingestion of the KE compared to 45 min after ingestion of the placebo (*p* = 0.005; d_z_: −0.84; d^95%CI^: −1.40, −0.25). Significant condition by time interactions were observed for CarbOx (Figure 3l; *p* = 0.040; *n* ^2^_p_ = 0.23), where CarbOx increased compared to baseline at 45 (*p* = 0.010; d_z_: 0.74; d^95%CI^: 0.17, 1.28) and 105 min (*p* = 0.001; d_z_: 1.03; d^95%CI^: 0.40, 1.62) following ingestion of the KE. CarbOx was also higher 45 min after ingestion of the KE compared to 45 min after ingestion of the placebo (*p* = 0.032; d_z_: 0.61; d^95%CI^: 0.06, 1.13), and 105 min after ingestion of the KE compared to 45 min after ingestion of the placebo condition (*p* = 0.006; d_z_: 0.82; d^95%CI^: 0.24, 1.38). There were no significant between‐group differences or interactions for RER (all *p* ≥ 0.075), CarbOx (all *p* ≥ 0.362), or FatOx (all *p* ≥ 0.128).

## 4 DISCUSSION

This study demonstrated that acute hyperketonemia achieved through the ingestion of oral KE significantly altered substrate utilization, evident by increased RER and CarbOx and decreased FatOx independent of MetS status. These findings support the hypothesis that exogenous ketosis can acutely shift substrate preference toward carbohydrate metabolism, even in individuals with metabolic impairments.

Our observed increase in RER following KE ingestion indicates a shift toward greater reliance on carbohydrate metabolism, which was further supported by the significant increase in CarbOx, concurrent reduction in FatOx, and decrease in blood glucose. These metabolic shifts occurred without significant changes in REE, suggesting that the effects of KE are substrate‐specific rather than reflective of overall energy expenditure changes. Interestingly, these effects were consistent across both MetS and non‐MetS groups, suggesting that the metabolic benefits of KE ingestion are not dependent on the presence of metabolic dysfunction. These findings are particularly relevant given that individuals with MetS often exhibit impaired metabolic flexibility (Smith et al., 2018), compounded by lower baseline FatOx rates, which may reflect underlying mitochondrial inefficiencies or insulin resistance. In this context, our observation of higher CarbOx and reduced blood glucose following KE ingestion further supports the metabolic utility of exogenous ketosis and aligns with previous research showing that KE can lower blood glucose and enhance indices of insulin sensitivity (Stubbs et al., 2017). Given that ketones are commonly associated with fat metabolism, our findings of increased CarbOx and reduced FatOx may appear counterintuitive. However, this metabolic shift can be explained by several βHB‐driven mechanisms, such as the preferential oxidation of ketones as an efficient fuel source (Cox et al., 2016), mild insulin‐mediated suppression of lipolysis (Zhao et al., 2020), and improved glucose uptake (Holdsworth et al., 2017; Myette‐Côté et al., 2019), all of which contribute to a transient shift away from FatOx. Specifically, βHB directly suppresses lipolysis via the HCAR2 receptor on adipocytes (Taggart et al., 2005), thereby reducing the availability of non‐esterified fatty acids for oxidation. Directly, βHB also stimulates insulin secretion from pancreatic β‐cells (Falkenhain et al., 2022), which may improve glucose uptake, while additionally lowering blood glucose indirectly through reduced hepatic glucose mobilization (Falkenhain et al., 2023; Reaven, 2005). Aligning with prior reports, we observed higher circulating βHB concentrations in individuals with MetS compared to those without MetS, which likely reflects the reduced ketone clearance commonly observed in those with impaired glucose metabolism (Sherwin et al., 1976). Despite this reduced clearance and utilization, blood glucose still decreased following KE ingestion in both groups, which suggests the role of other insulin‐independent mechanisms such as delayed intestinal glucose absorption and/or gut‐derived hormones (Falkenhain et al., 2023). Collectively, these findings indicate that KE ingestion lowers blood glucose and is associated with a greater reliance on CarbOx; however, the observed increase in CarbOx should be interpreted with caution, as this may reflect changes in relative substrate utilization that rose secondary to suppressed FatOx, rather than changes in absolute substrate flux. Additional explanations for our findings are presented hereafter.

Upon ingestion, ketone bodies such as βHB are rapidly absorbed (evident by our findings for βHB) and oxidized by metabolically active tissues (i.e., brain, heart, and skeletal muscle), effectively serving as an alternative to fatty acids for energy production (Cox et al., 2016). Because ketones yield more energy per unit oxygen than fatty acids (Cotter et al., 2013), making them a more oxygen‐efficient substrate, their availability reduces the need for FatOx, particularly under resting or low‐intensity conditions. As a result, the body may transiently prioritize ketone and glucose oxidation, leading to a reduced reliance on fat as an energy source. In individuals with MetS, who often exhibit mitochondrial dysfunction and lower baseline FatOx (Kim et al., 2000), the provision of a more metabolically efficient fuel source such as KE may help compensate for impaired metabolic function by improving energy availability and facilitating more effective carbohydrate utilization.

Although KE contain little to no carbohydrate, they can elicit a mild insulin response. This aligns with previous studies reporting modest increases in insulin following KE or ketogenic meal ingestion (Graybeal et al., 2021; Stubbs et al., 2017), which may suppress lipolysis and enhance glucose uptake and oxidation (Kelley et al., 1999); consistent with prior findings of reduced postprandial lipid concentrations following a ketogenic meal (Graybeal, Kreutzer, Moss, & Shah, 2024), as well as our findings of reduced FatOx and glucose after KE consumption. In the context of MetS, where insulin resistance impairs glucose uptake, KE may help bypass this limitation by providing an alternative energy substrate with the capacity to indirectly enhance glucose transporter activity (Yu et al., 2016; Zhang et al., 2025), potentially contributing to our observed reductions in blood glucose and increases in CarbOx. These findings may also be partially explained by mechanisms of homeostatic energy regulation. Ghrelin, a peripheral appetite‐stimulating hormone, acts as a counter‐regulatory signal during fasting by suppressing insulin secretion from pancreatic β‐cells and promoting hepatic glucose production to maintain blood glucose levels (Poher et al., 2018). Studies have shown that ingestion of KE or ketogenic meals reduces circulating ghrelin in parallel with reductions in blood glucose and modest increases in insulin (Graybeal et al., 2021; Stubbs et al., 2017; Stubbs et al., 2018). While reduced ghrelin and increased insulin typically result in increased REE based on classical energy balance principles (St‐Pierre et al., 2004), our findings could indicate otherwise. Specifically, we observed increased CarbOx and lower blood glucose following KE ingestion without corresponding increases in REE. This suggests that KE ingestion does not necessarily upregulate total energy metabolism, but may instead shift substrate utilization toward greater CarbOx. This is potentially due to improved insulin sensitivity and secretion, which could be partially facilitated by the suppression of ghrelin following KE ingestion.

Despite the potential for our findings to be explained by the aforementioned physiological mechanisms, it is also important to recognize the methodological limitations of interpreting RER in the presence of exogenous ketones. Specifically, βHB oxidation yields an RER of ~0.89, which more closely resembles the profile of CarbOx (1.00) than that of FatOx (0.71) (Livesey & Elia, 1988), suggesting that increases in RER following exogenous ketone ingestion may be driven, at least in part, by ketone oxidation itself rather than a true shift toward greater CarbOx. Under normal endogenous ketosis, where the body produces ketones in the absence of carbohydrates, RER typically drops alongside increases in FatOx. However, the ingestion of ketones presents a unique metabolic challenge, where the presence of exogenous ketones without a reduction in circulating carbohydrates makes the interpretation of RER more complex, as the measured value may reflect a combination of carbohydrate, fat, and ketone oxidation. In our study, the concurrent decrease in blood glucose and increase in circulating βHB suggests that exogenous ingestion may have contributed to changes in substrate utilization; however, we cannot entirely conclude CarbOx from βHB oxidation using indirect calorimetry alone. Therefore, while the observed rise in RER may reflect a meaningful shift in metabolism, it should be interpreted cautiously, acknowledging that RER values in the presence of ketones partly reflect the stoichiometric properties of βHB oxidation and may not exclusively indicate increased CarbOx. Interestingly, we also observed that participants without MetS exhibited a small increase in circulating βHB during the placebo condition (i.e., fasting), indicating an ability to mobilize and utilize fat‐derived substrates during periods with low available energy. Conversely, this response was absent in the MetS group, who maintained a higher RER, consistent with reliance on CarbOx even under fasting‐like conditions. This may suggest a reduced capacity in MetS to shift toward fat‐derived fuel use in metabolic situations that would typically favor fatty acid‐derived ketone production.

There are several limitations of the current study that warrant discussion. First, investigators were not blinded to treatment conditions; however, single‐blind designs are commonly employed when relative dosing is required and when placebo formulations must be matched for appearance, volume, and taste independent of the manufacturer. Circulating insulin and free fatty acid concentrations were not measured in this study, limiting our ability to draw direct conclusions about insulin and lipid responses. However, these responses to KE ingestion are well‐documented (Veech, 2014), and based on our findings, it is reasonable to expect that insulin and lipid profiles followed patterns consistent with those reported in previous research (Stubbs et al., 2017; Veech, 2014). Although the KE and placebo treatments were not energy‐matched, their caloric content was minimal relative to participants' total daily energy requirements (average KE: 125 kcal for the MetS group; 90.8 kcal for the non‐MetS group). This is supported by prior studies showing no change in postprandial REE following similar test meal sizes (~100 kcals) in normal weight participants (Martin et al., 2000). Thus, the minor differences in energy content between conditions likely had minimal, if any, impact on our findings, as further supported by the absence of differences in REE across treatments (Graybeal et al., 2025). Our study sample included participants with MetS who were prescribed medications to manage MetS‐related risk factors, which may have influenced metabolic measurements. However, these non‐insulin pharmacological treatments are commonly used for such conditions, and participants refrained from taking their medications on the morning of their study visits. While our study utilized a modest sample size, a notable strength was the matching of MetS and non‐MetS participants across multiple racial and ethnic groups, as well as the inclusion of both male and female individuals. Finally, respiratory gases were collected for only a short period. While the short duration of respiratory gas collection limits conclusions about long‐term metabolic adaptations, the acute shifts in substrate utilization observed here provide a foundation for future investigations. Longitudinal studies are needed to determine whether repeated KE administration can lead to sustained improvements in metabolic flexibility and glycemic control, particularly in individuals with MetS. Although acute respiratory gas exchange responses to KE ingestion have not been previously examined in individuals with MetS, investigating these effects represents a critical milestone prior to pursuing long‐term interventions.

## 5 CONCLUSION

In conclusion, oral KE ingestion acutely increases CarbOx and RER while reducing FatOx, independent of metabolic health status. These findings highlight the potential of hyperketonemia via exogenous ketosis as a non‐pharmacological metabolic therapy in individuals with or at risk for MetS. The ability of KE to acutely enhance CarbOx in this population may offer a novel strategy to improve metabolic efficiency and glycemic control.

## AUTHOR CONTRIBUTIONS

Austin J. Graybeal and Jon Stavres designed, supervised, and provided resources for the study and RP validated and edited the manuscript. Caleb F. Brandner, Ryan S. Aultman, Anabelle Vallecillo‐Bustos, Abby T. Compton, Sydney H. Swafford, and Ta'Quoris A. Newsome collected/compiled the data. Austin J. Graybeal analyzed the data and wrote the manuscript and all authors reviewed/edited and approved the manuscript.

## FUNDING INFORMATION

This work was also supported, in part, by the National Institute of General Medical Sciences of the National Institutes of Health under grant number NOT‐GM‐23‐034 (FAIN# U54GM115428). The funding agency had no role in the study design, collection, analysis or interpretation of the data, writing the manuscript, or the decision to submit the paper for publication.

## CONFLICT OF INTEREST STATEMENT

The authors declare no conflicts of interest.

## ARTIFICIAL INTELLIGENCE GENERATED CONTENT (AIGC)

Generative artificial intelligence tools were not used in the preparation of this manuscript. Tools were used solely to improve spelling, grammar or general editing, but these are not included within the publisher's scope.

## CODE AVAILABILITY STATEMENT

Code is available upon reasonable request to the authors.

## ETHICS STATEMENT

The study procedures were conducted according to the ethical standards laid down in the Declaration of Helsinki and all procedures involving human subjects/patients were approved by the University of Southern Mississippi Institutional Review Board (IRB# 24‐0476). Written informed consent was obtained from all participants prior to participation.

## Data Availability

Data are available upon reasonable request to the authors.
